# Pectoral-Intercostal Fascial Plane Block for the Management of Resistant Chronic Post-sternotomy Pain Syndrome

**DOI:** 10.7759/cureus.107423

**Published:** 2026-04-20

**Authors:** Rajat Shuvra Das, Muhammad Nasif Imtiaz, Khan Md. Amanur Rahman, Sanjoy Kumar Saha

**Affiliations:** 1 Department of Anaesthesia, Analgesia and Intensive Care Medicine, Bangladesh Medical University, Dhaka, BGD; 2 Department of Cardiac Surgery, Bangladesh Medical University, Dhaka, BGD

**Keywords:** chronic pain management, interventional pain medicine, multimodal analgesia, pectoral-intercostal fascial plane block, post-sternotomy pain syndrome

## Abstract

Chronic post-sternotomy pain syndrome (PSPS) is a common complication following cardiac surgery performed through a median sternotomy. Persistent parasternal pain often resists standard multimodal pharmacological therapy and impacts quality of life. The pectoral-intercostal fascial plane block (PIFB) is an ultrasound-guided regional anesthesia technique targeting the anterior cutaneous branches of the thoracic intercostal nerves. It may provide adequate analgesia in post-sternotomy pain. A 59-year-old man presented with severe parasternal and chest wall pain five months after coronary bypass surgery. The pain was sharp, burning, and tingling, worse at night and with movement. Examination showed parasternal tenderness with allodynia. The numerical rating scale (NRS) score was 8/10, and the PainDETECT score was 17/35, indicating neuropathic pain. After six weeks of conservative treatment, pain relief was 30%. An ultrasound-guided bilateral PIFB was performed. The patient's NRS score decreased to 1/10 post-procedure. At one- and three-month follow-up, he reported 80% pain relief and improved sleep and function, without complications. This case highlights ultrasound-guided PIFB as an effective intervention for resistant chronic PSPS. Given its superficial approach, simplicity, and safety profile, PIFB may be a valuable adjunct to multimodal pain management in selected patients and merits further evaluation in larger studies.

## Introduction

Chronic post-sternotomy pain syndrome (PSPS) is a debilitating complication affecting 14-56% of patients after cardiac surgery via median sternotomy [1]. Defined as pain persisting beyond the expected healing period, typically three months, chronic post-operative pain can severely impact quality of life, leading to sleep disturbances, limitations in daily activities, and adverse effects on cardiovascular and pulmonary function [2]. Despite advances in surgical techniques and acute pain management, a substantial proportion of patients who undergo sternotomy for cardiac surgery report persistent pain [3]. The management of PSPS is complex and often necessitates a multidisciplinary approach that integrates pharmacological, non-pharmacological, and interventional strategies [4]. In this case report, we successfully managed a patient with chronic PSPS following off-pump coronary artery bypass (OPCAB) surgery using a pectoral-intercostal fascial plane block (PIFB).

## Case presentation

A 59-year-old man was referred to our pain clinic for severe parasternal and surrounding pain, five months after a coronary artery bypass graft. He described sharp, tingling, and burning pain that worsened at night. The patient was pain-free for 1.5 months post-surgery. He received oral paracetamol, diclofenac sodium, and tramadol from the cardiac surgeons before referral. Examination revealed parasternal tenderness with allodynia but no hyperalgesia. Pain increased during coughing and straining during defecation. His numerical rating scale (NRS) score was 8/10, and the pain detection score was 17/35. Chest radiography was normal, showing a sternal wire in situ. We diagnosed the patient with chronic PSPS. We started oral pregabalin 50 mg daily, titrated to 100 mg, then added oral duloxetine 20 mg daily, titrated to 60 mg. He received Oral Ibuprofen 200 mg twice daily and paracetamol 500 mg thrice daily for 10 days, plus 0.025% capsaicin ointment four times daily. After six weeks, he reported 30% pain relief. The NRS score was 6/10, and the pain detection score was 12/35. Owing to ongoing parasternal pain, a PIFB was considered and agreed upon by the patient. The patient was on aspirin and clopidogrel 75 mg each. After obtaining written consent, the patient was prepared and placed in the supine position under aseptic precautions.

**Figure 1:**
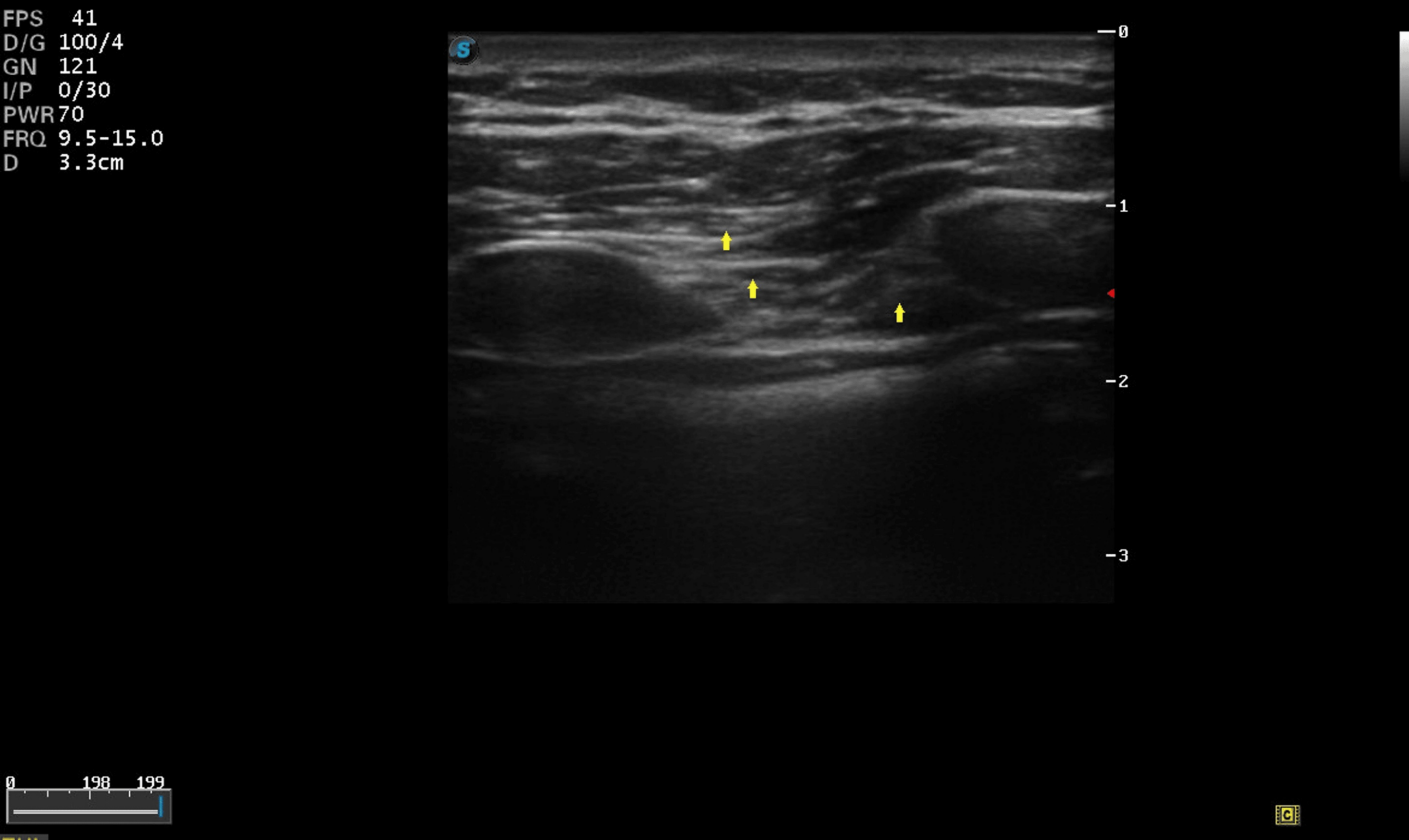
Arrows from above to below indicate the pectoralis muscle, intercostal muscles and transversus thoracis muscle. The probe was placed at 3 cm lateral to the midline on the right side of the chest.

A linear high-frequency probe was placed 3 cm lateral to the mid-sternal line on the right side (Figure 1).

**Figure 2:**
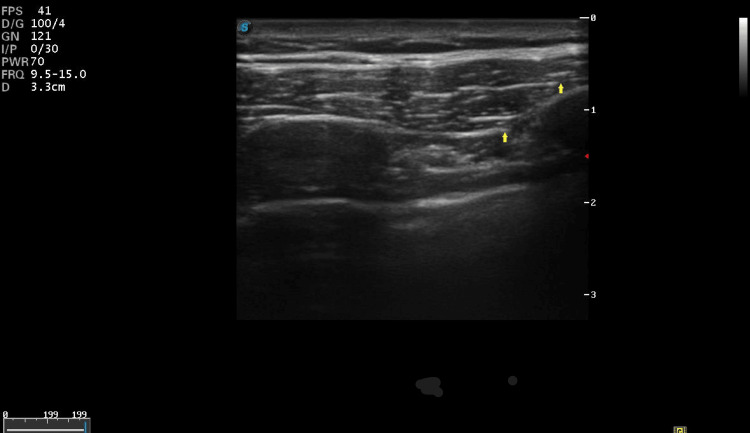
Arrows indicate needle trajectory with the tip between the pectoralis muscle and intercostal muscle, with the probe position same as before.

A 10 cm 22 G echogenic needle was inserted into the 2nd-3rd intercostal space from caudal to cephalad, and 10 ml of drug mixture was injected between the pectoralis major and external intercostal muscles (Figure 2).

A second injection was administered in the 4th-5th intercostal space. Both injections were repeated on the right side using 40 ml of 0.0625% bupivacaine with 8 mg dexamethasone. The post-procedure NRS was 1/10. He was discharged after one hour with oral pregabalin 50 mg twice daily, duloxetine 30 mg twice daily, and paracetamol 500 mg thrice daily. He had 80% pain relief and was fully satisfied at both one- and three-month follow-ups.

## Discussion

The PIFB is a relatively new regional technique that has gained widespread use. It is a fascial plane block that involves the deposition of local anesthetic between two superficial muscles: the pectoralis major and the external intercostal [5]. This involves the anterior cutaneous branches of the thoracic intercostal nerves (T2-T6), which innervate the sternum and surrounding parasternal area [6]. Historically, PIFB was first described for analgesia of the sternal aspect following breast surgery [7]. Its utility has expanded to include the management of acute post-sternotomy pain following cardiac surgery.

Diagnosing PSPS requires systematic exclusion of other causes of chest pain, particularly active cardiac disease, sternal instability, and mediastinitis [8]. In our case, the cardiac surgeon confirmed the absence of cardiac pathology. Management often involves pharmacological agents like opioids and paracetamol or neuropathic medications such as pregabalin [9]. PIFB can be used as an adjunct to multimodal analgesia for treating PSPB while maintaining patient safety. Unlike other regional techniques, it is a superficial, simpler block near the incision site, away from major vascular structures, highly reproducible, and associated with a low complication rate [10]. Pain reduction after cardiac surgery indicates the effectiveness of treatment. Long-term pain management is crucial for improving patients' quality of life and functional outcomes.

This case demonstrates PIBF's potential in managing chronic PSPS after OPCAB surgery when conventional treatments fail. In this case, the marked pain relief achieved with the PIFB suggests a promising role for this technique in the management of chronic PSPS, meriting further study.

## Conclusions

This case highlights the potential role of ultrasound-guided PIFB as an effective and safe intervention for resistant chronic PSPS. Given its superficial approach, technical simplicity, and favorable safety profile, PIFB may be a valuable adjunct to multimodal pain management in selected patients and merits further evaluation in larger clinical studies.

## References

[1] McGillion MH, Henry S, Busse JW (2019). Examination of psychological risk factors for chronic pain following cardiac surgery: protocol for a prospective observational study. BMJ Open.

[2] Zubrzycki M, Liebold A, Skrabal C, Reinelt H, Ziegler M, Perdas E, Zubrzycka M (2018). Assessment and pathophysiology of pain in cardiac surgery. J Pain Res.

[3] Kleiman AM, Sanders DT, Nemergut EC, Huffmyer JL (2017). Chronic poststernotomy pain: incidence, risk factors, treatment, prevention, and the anesthesiologist’s role. Reg Anesth Pain Med.

[4] Dost B, Turunc E, Aydin ME (2025). Pain management in minimally invasive cardiac surgery: a review of current clinical evidence. Pain Ther.

[5] Zhao Y, He D, Zhou W (2025). Effects of continuous pecto-intercostal fascial block for management of post-sternotomy pain in patients undergoing cardiac surgery: a randomized controlled trial. Int J Surg.

[6] Sahoo RK, Kar R, Patel R, Kumar M, Giri D, Biswas M, Nair AS (2022). Pectoral-intercostal fascial plane block in chronic post-sternotomy pain. Ann Card Anaesth.

[7] de la Torre PA, García PD, Alvarez SL, Miguel FJ, Pérez MF (2014). A novel ultrasound-guided block: a promising alternative for breast analgesia. Aesthet Surg J.

[8] Bordoni B, Marelli F, Morabito B, Sacconi B, Severino P (2017). Post-sternotomy pain syndrome following cardiac surgery: case report. J Pain Res.

[9] Hamaguchi S (2020). Treatment of chronic postsurgical pain in pain medicine. J Jpn Soc Clin Anesth.

[10] Joshi P, Borde D, Apsingekar P, Pande S, Tandale M, Deodhar A, Jangle S (2024). Pecto-intercostal fascial plane block: a novel technique for analgesia in patients with sternal dehiscence. Ann Card Anaesth.

